# Autoinflammatory syndromes of STING and TREX1 dysfunction

**DOI:** 10.1172/JCI204549

**Published:** 2026-06-01

**Authors:** Debby J. Park, Kate M. Jones, Jessica B. Anderson, Amanda V. Finck, Jonathan J. Miner

**Affiliations:** 1 RVCL Research Center, Department of Medicine;; 2 Department of Microbiology;; 3 Institute for Immunology and Immune Health; and; 4 Colton Center for Autoimmunity, University of Pennsylvania Perelman School of Medicine, Philadelphia, Pennsylvania, USA.

## Abstract

Breakthroughs in rare genetic disease research elucidate the relationships among cytosolic DNA sensing, genome instability, and autoimmune disease phenotypes. Cytosolic self-DNA is a potent trigger of innate immunity, activating the DNA sensor cyclic GMP-AMP synthase (cGAS) and its downstream effector stimulator of interferon genes (STING). This pathway is negatively regulated by the DNA-degrading enzyme three-prime repair exonuclease 1 (TREX1); loss-of-function *TREX1* variants lead to accumulation of cytosolic DNA, resulting in STING-mediated autoinflammation. Similarly, STING gain-of-function mutations cause STING-associated vasculopathy with onset in infancy, another disease characterized by multi-organ damage, disability, and premature death. The TREX1-cGAS-STING pathway has also been implicated in regulation of genome stability. Indeed, DNA damage lies at the heart of a separate TREX1-mediated disease, known as retinal vasculopathy with cerebral leukoencephalopathy, where the aberrant nuclear activity of mislocalized TREX1 damages genomic DNA, resulting in multi-organ degeneration syndrome with features of autoimmunity. Thus, monogenic autoimmune diseases and DNA damage syndromes sometimes overlap clinically, and the study of these diseases has created pathways for developing first-in-class small molecule therapeutics.

## Introduction

Rare inflammatory diseases caused by single-gene mutations in nucleic acid–sensing pathways present a unique view into disease etiologies and fundamental biological principles. In particular, key insights into disease mechanisms have been gleaned from the study of STING-associated vasculopathy with onset in infancy (SAVI), caused by stimulator of interferon genes 1 (*STING1*) variants (1), as well as Aicardi-Goutières syndrome (AGS) (2), retinal vasculopathy with cerebral leukoencephalopathy (RVCL or RVCL-S) (3, 4), and familial chilblain lupus (FCL) (5), all linked to three-prime repair exonuclease 1 (*TREX1*) variants. Here, we review how these genetic diseases have elucidated the role of TREX1, cyclic GMP-AMP synthase (cGAS), and STING in both common and rare autoimmune and autoinflammatory diseases. For example, cGAS and STING have been implicated in classical autoimmune diseases, such as systemic lupus erythematosus (SLE) and rheumatoid arthritis (6), as well as syndromes that share clinical features with SAVI, including systemic sclerosis (7) and dermatomyositis (8, 9). In addition to regulating inflammation, TREX1, cGAS, and STING are now linked to cellular senescence as part of the DNA damage theory of aging (10–12). Here, we propose a related DNA damage theory of autoimmunity and autoinflammation.

## TREX1, cGAS, and STING in monogenic autoinflammation

**Figure 1:**
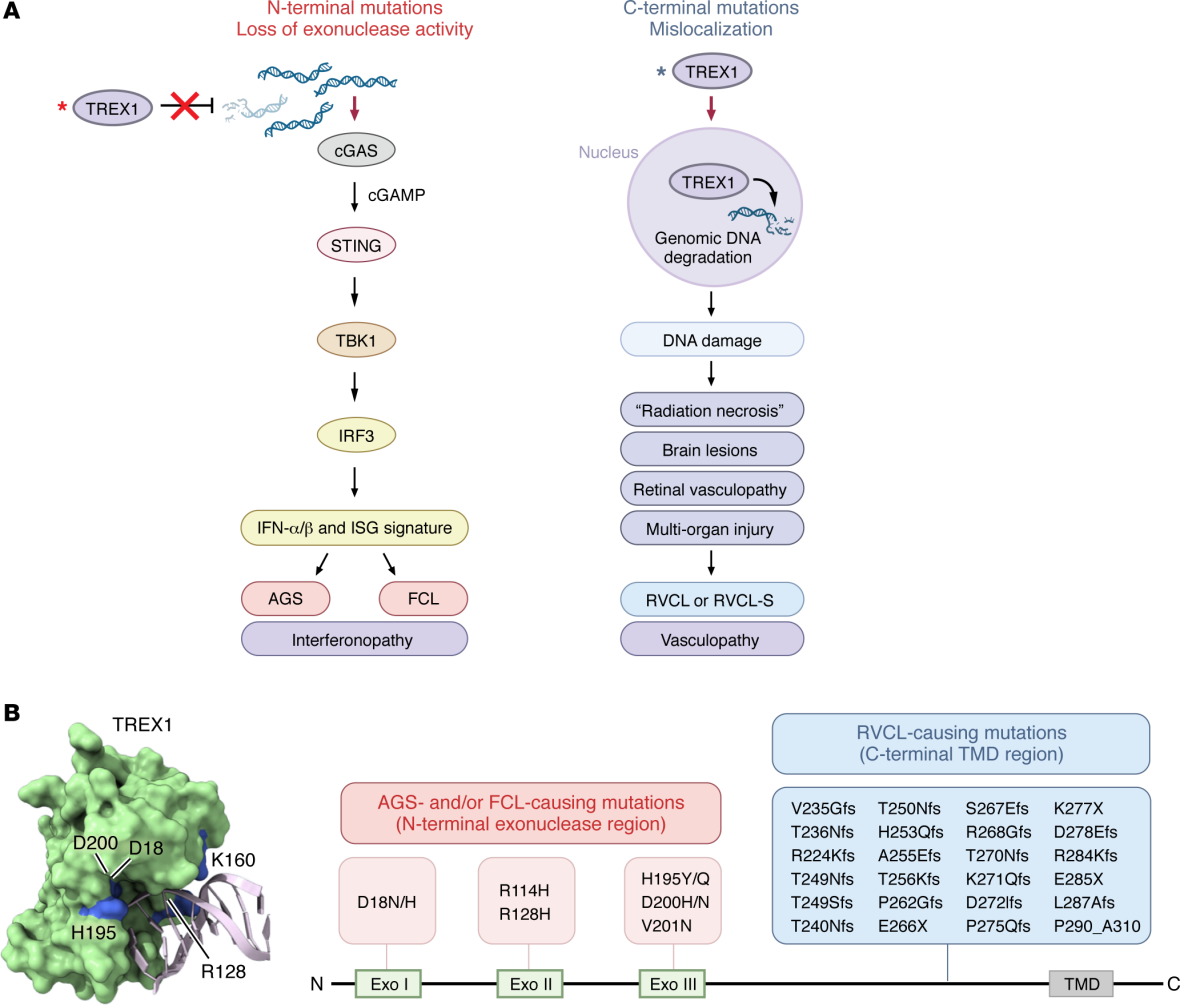
N-terminal mutations in TREX1 cause multi-organ inflammatory diseases, and C-terminal mutations cause DNA damage with multi-organ injury and features of autoimmunity. (**A**) Loss of TREX1 exonuclease activity triggers accumulation of cytosolic DNA, unabated STING activation, and interferonopathy in AGS and FCL. By contrast, C-terminal mutations in TREX1 lead to autosomal-dominant RVCL, a multi-organ vasculopathy associated with genome instability and organ degeneration, often mimicking common autoimmune diseases, with onset around midlife and universal disability and premature mortality. Asterisks indicate mutant protein. (**B**) TREX1 (shown as a monomer) crystal structure reveals AGS- and FCL-associated mutations (shown in blue) that are located at the DNA (shown in gray) interface and lead to a loss of proper DNA binding or catalytic activity. Truncating mutations on the C-terminus associated with RVCL lead to a loss of the transmembrane domain (TMD; not indicated in structure) with localization to the nucleus, triggering DNA damage, cellular senescence, and apoptosis. Structure visualized using UCSF ChimeraX (177). TBK1, tank binding kinase 1; ISG, interferon-stimulated gene; IRF3, interferon regulatory factor 3; cGAMP, cyclic GMP-AMP.

**Figure 2:**
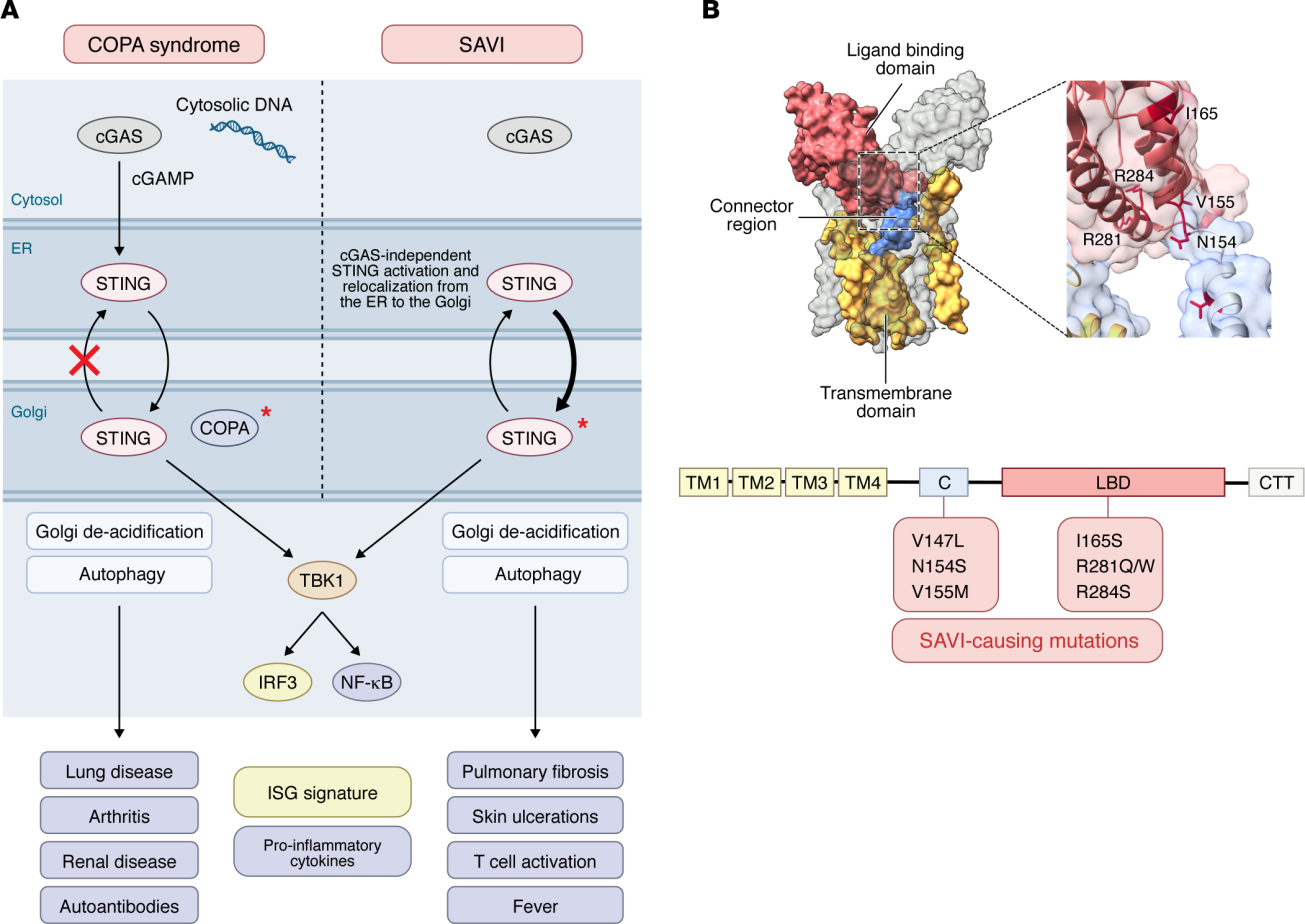
Spontaneous activation of STING occurs with disease-causing STING gain-of-function mutations, as well as with non-STING mutations that cause accumulation of STING in the Golgi. (**A**) Coatomer protein complex subunit A (COPA) mutations reduce STING retrieval from the Golgi, leading to constitutive WT STING activation and systemic autoimmunity in patients with COPA syndrome. By contrast, STING gain-of-function mutations in SAVI lead to ligand-independent STING activation and multi-organ autoinflammation. Asterisks indicate mutant protein. (**B**) SAVI-causing STING mutations primarily involve the connector loop and polymer interface portion of the ligand-binding domain (LBD). Apo STING (shown as a dimer) crystal structure consists of four transmembrane (TM) helices that make up the N-terminal transmembrane domain (TMD; yellow), connector region (blue), LBD (pink), and C-terminal tail (CTT; not indicated in structure). STING is localized in the endoplasmic reticulum prior to its activation and trafficking to other cellular compartments. The connector region links the TMD to the LBD, where cGAMP binds, which represents the genetic location of SAVI gain-of-function mutations. Structure visualized using UCSF ChimeraX (177).

Monogenic diseases are especially helpful in understanding the unique contributions of TREX1, cGAS, and STING to human disease (1, 2, 4, 5, 13–15) (Figures 1 and 2 and Table 1). Regarding *TREX1* variants, the associated disease phenotypes are best differentiated by the domain in which the mutation occurs (2, 3, 5). N-terminal TREX1 mutants affect the catalytic domain, diminishing or eliminating exonuclease activity. This results in accumulation of cytosolic DNA and activation of the cGAS-STING pathway (16, 17). The most severely disruptive variants cause AGS, a syndrome associated with a variety of mutations in genes regulating nucleic acid sensing, including *TREX1* (18–23). These genetic variants activate the type I IFN response, leading to progressive cognitive impairment and other neurological deficits as well as systemic manifestations (22, 24, 25). In addition to hypercytokinemia, patients with AGS can develop autoantibodies, including autoantibodies associated with SLE (22, 26). Autoantibodies in AGS might result from type I IFN or, alternatively, immunostimulatory cellular debris (27, 28). Some patients with AGS develop autoimmune manifestations, including immune thrombocytopenia and autoimmune thyroiditis (22, 29, 30) (Table 1). Unlike humans with AGS, who develop neuroinflammation, TREX1 deficiency in mice leads to cardiovascular inflammation, a distinction that might reflect species-specific differences in cGAS-STING activation. Genetic deletion of *Cgas*, *Sting1*, or the type I IFN receptor (*Ifnar1*) abrogates this disease-associated lethality in mice (13, 16, 17, 31, 32). Furthermore, elimination of T and B cells is protective in *Trex1^–/–^* animals (17), supporting a combined role of autoimmunity and autoinflammation.

Some *TREX1* variants in the N-terminal catalytic domain cause a disease distinct from AGS, known as FCL (5, 33). FCL is characterized by painful, cold-induced acral lesions, along with a variety of systemic manifestations that can include cerebrovascular disease and elevated type I IFN in the peripheral blood (5, 33–35). Generally, FCL is associated with mutations that cause a milder reduction in exonuclease activity compared with the profound abrogation of TREX1 activity in AGS (2, 5, 33) (Table 1). However, the existence of identical point mutations that can cause either AGS or FCL suggests that these diseases exist on a spectrum (5, 36–38). For example, TREX1 D18N is a dominant-negative mutation that requires only one allele to cause FCL (33, 39), but it can also cause more extreme AGS-associated pathology, even within the same family (36).

Unlike the exonuclease domain TREX1 mutants that cause AGS and FCL, C-terminal truncating mutations proximal to the TMD produce a mislocalized TREX1 that retains highly potent exonuclease activity and freely distributes throughout the cytosol and nucleus (3). This gives rise to a distinct clinical syndrome known as RVCL or RVCL-S (3, 4), previously called HERNS (40). RVCL manifests in adulthood with severe small vessel disease, leading to retinal, kidney, and brain injury and eventual death in 5–10 years (3, 15) (Table 1). Intriguingly, RVCL does not typically present with any appreciable systemic inflammation, though the associated organ damage mimics that of common autoimmune diseases (41, 42). This helps explain why RVCL is often confused with inflammatory conditions like MS, central nervous system (CNS) vasculitis, SLE, and neurosarcoidosis (3, 15, 41, 43–45). However, unlike those inflammatory diseases, RVCL appears to be a DNA damage syndrome caused by mislocalization of the truncated TREX1 protein to the nucleus resulting in genomic instability (4) (Figure 1). This underscores the clinical overlap between autoimmunity and DNA damage syndromes. RVCL-associated cellular damage is ameliorated by catalytically inactive TREX1 mutants, implicating exonuclease activity in RVCL pathogenesis (4). Nevertheless, cytokines may still play a role in RVCL, since TREX1 itself is an ISG (46, 47). Indeed, transcriptional upregulation of the TREX1 mutant during aging might explain late onset of symptoms in RVCL (4). Thus, C-terminal TREX1 truncation results in RVCL, a consequence of gain-of-function TREX1 nuclear mislocalization, which creates a completely distinct disease from AGS.

In contrast with human TREX1 deficiency, diseases of human cGAS or STING deficiency have not yet been described. However, mice lacking cGAS and STING are born at normal Mendelian ratios, do not exhibit gross developmental defects, and are healthy in barrier facilities (48–50). These mice are more vulnerable to infection with RNA and DNA viruses than WT animals, due to impaired innate and adaptive immunity (48–51). Genetic deletion of cGAS in mice leads to impaired induction of cellular senescence and defective antitumor immunity, though spontaneous tumor development is less impacted (52, 53). Besides its role in antiviral and antitumoral immunity in mice, the cGAS-STING pathway also contributes to normal aging. Indeed, older adult mice deficient in STING exhibit reduced levels of inflammation and neurodegeneration (54), and cGAS also has been implicated in longevity in studies of the naked mole rat (10). Thus, the cGAS-STING pathway is a potential therapeutic target for autoimmune and autoinflammatory diseases as well as age-related neurodegenerative diseases. Importantly, genetic deficiency of cGAS and STING do not phenocopy each other (55), which might be explained in part by the recent discovery of PIP(3,5)P_2_ as a cofactor for STING activation (56).

SAVI-associated STING gain-of-function mutants result in conformational changes and spontaneous translocation of STING from the ER to the Golgi (1, 57). These mutations cluster primarily in the connector loop and the polymeric interface of the LBD, promoting ligand-independent polymerization and constitutive activation of the pathway (58). This hyperactivation of STING clinically manifests as cutaneous vasculopathy, interstitial lung disease (ILD), and systemic inflammation (1) (Table 1). While the CNS is not typically considered a primary target organ in SAVI, rare cases of CNS involvement have been reported, including cerebral vasculitis, brain infarction, and white matter lesions, suggesting that the CNS is affected in a subset of patients (59, 60). Vascular injury, reflected by Raynaud’s phenomenon, cutaneous ulcers, and retinal vasculopathy, is among the most disabling features of SAVI (Table 1). Patients with SAVI also frequently develop T cell cytopenia, hypergammaglobulinemia, and leukopenia (Table 1), suggesting that chronic STING-driven innate immune activation secondarily dysregulates the adaptive immune compartment (1, 61). Indeed, some patients with SAVI have SLE-associated autoantibodies (61).

Whereas in SAVI, conformational changes in STING lead to spontaneous translocation of mutant STING to the Golgi, Golgi mislocalization of WT STING also leads to spontaneous signaling and systemic autoimmunity, albeit with distinct clinical phenotypes from SAVI. For example, COPA syndrome results from failure of WT STING to be retrieved from the Golgi, triggering persistent signaling (62–64) (Figure 2). Compelling genetic evidence for this mechanism comes from a recent study showing that COPA mutation–carrying patients who also have the hypomorphic STING HAQ polymorphism are protected from disease (65). This observation suggests that fully functional WT STING is necessary for COPA pathology. It seems likely that additional STING polymorphisms may also regulate disease severity or penetrance in the context of other autoimmune and autoinflammatory diseases (66, 67).

## IFN-independent functions of STING in autoinflammatory disease

Type I interferonopathies are characterized by upregulation of ISGs, driven by production of type I IFNs as a consequence of constitutive signaling through STING or other innate adaptors. In syndromes of chronic STING activation, such as SAVI and AGS, the IFN signature is associated with inflammatory tissue damage. However, if type I IFN and ISGs were the sole pathogenic drivers, one would expect that pharmacological blockade of JAK/STAT would prevent organ damage. In practice, JAK inhibitors only partially ameliorate AGS- and SAVI-associated clinical symptoms (68, 69). The largest open-label study of a JAK1/2 inhibitor in AGS reported improvements in clinical scores, skin inflammation, and developmental milestones, but long-term protection against neurological progression remains uncertain (69). In patients with SAVI, baricitinib and tofacitinib can produce dramatic healing of cutaneous ulcers, yet ILD frequently progresses despite treatment, representing a key limitation of JAK inhibition (68, 70). This suggests additional IFN-independent mechanisms of SAVI pathogenesis. These may include STING-mediated regulation of Golgi pH (71, 72), activation of noncanonical autophagy (72), and promotion of lysosomal biogenesis through activation of the master transcriptional regulator, transcription factor EB (73).

To understand SAVI pathology, we must also consider functions of STING beyond cytokine induction. The primordial function of STING is believed to be that of an autophagy inducer (74). Upon cGAMP binding, STING translocates to the ER-Golgi intermediate compartment, initiating LC3-mediated lipidation and promoting noncanonical autophagy, even in the setting of TBK1 inhibition (75). In fact, this autophagic function is shared by the STING orthologs in *Xenopus* and sea anemone *Nematostella vectensis*, two species that lack STING C-terminal motifs required for TBK1 and IRF3 activation (74). The characterization of STING as a proton channel has resulted in a model of proton-triggered lipidation and autophagy induction, which may also account for STING’s ability to activate the NLRP3 inflammasome (75, 76).

Despite SAVI being classified as a type I interferonopathy in humans (1), mouse models have revealed that nearly all the major disease manifestations develop independently of the downstream effectors IRF3, IRF7, and IFNAR1 (77). Thus, STING activation is now understood to serve much broader cellular and immunological roles than were previously known. One of the first mouse models of SAVI, harboring the STING N153S gain-of-function mutation, exhibits lung inflammation, T cell cytopenia, and myeloid cell expansion (77). Surprisingly, lung pathology and immune dysfunction persist in the absence of IRF3, which is required for type I IFN induction downstream of STING. SAVI can also develop in *cGAS^–/–^* as well as in germ-free mice, independently of microbes and associated cyclic dinucleotide ligands (78). Extending these findings, SAVI mice have severe combined immunodeficiency disease, associated with T cell cytopenia, and none of these immunological phenotypes are prevented by genetic deletion of *Ifnar1* (79–82). Lymph node organogenesis and Treg differentiation are restored in SAVI mice lacking the type II IFN receptor, further supporting a role for type II over type I IFN in mice (83, 84). Endothelial cells also contribute to inflammatory lung disease in SAVI mice, with prominent roles for chemokines and TNF in promoting endothelial barrier disruption associated with leukocyte infiltration, reminiscent of tertiary lymphoid structures, into the lungs (85, 86). TNF also plays a role in neuroinflammation in SAVI animals (86). Thus, STING gain-of-function pathology extends beyond the IRF3-dependent type I IFN signaling.

Recently discovered regulatory mechanisms have yielded insights into subcellular compartment-specific functions of STING. One proposed role of STING is maintaining ER calcium homeostasis. For example, STING gain of function disrupts calcium stores, activates the unfolded protein response, and can promote T cell death via chronic ER stress (87). Separately, a genome-scale CRISPR screen using the STING gain-of-function mutant uncovered ADP-ribosylation factor GTPase-activating protein 2 (ArfGAP2) as a major regulator of STING in antigen-presenting cells (71). Genetic deletion of ArfGAP2 prevented STING-mediated proton channel activity and almost completely prevented SAVI-associated inflammatory disease in mice (71). This suggests that therapeutic targeting of the ArfGAP2-dependent trafficking pathway, rather than the canonical TBK1/IRF3 axis, could lead to novel therapies. Thus, noncanonical and cell type–specific functions of STING remain a major area of interest.

Given STING’s central role in responding to pathogens, some have suggested that viruses and other microbes might exacerbate SAVI. Indeed, introduction of a common murine γ-herpesvirus (γHV68) — closely related to Epstein-Barr virus and Kaposi sarcoma–associated herpesvirus — led to severe pulmonary fibrosis and rapid death in SAVI mice (88). By contrast, germ-free SAVI mice are unprotected from spontaneous autoinflammation and lung disease (78). Therefore, it is plausible that viruses contribute to ILD and fibrosis in patients with SAVI, though the hypothesis would be challenging to prove epidemiologically in this ultrarare disease.

In summary, the current picture of SAVI pathogenesis suggests that STING activation in multiple cell types can mediate disease through distinct effector arms. IFN-independent functions of STING — particularly its role in driving autophagy, regulating Golgi pH, and promoting NLRP3 inflammasome activation — may directly contribute to the lung inflammation of SAVI mice, as each of these pathways has been implicated during inflammatory signaling in other contexts. Adaptive immunity also plays a key role, based on the fact that *Rag1^–/–^* SAVI mice are almost completely protected from lung disease, unlike *Ifnar1^–/–^* SAVI mice, which are not protected at all (81). By contrast, deletion of the type II IFN receptor (IFNGR1) reduces lung inflammation and restores lymph node development (83). Finally, STING activation in nonhematopoietic cells, including endothelial cells, also likely contributes to disease (85). Further dissecting the cell type–specific contributions of chronic STING activity will require conditional knockout strategies.

## Sources and mechanisms of cytosolic DNA release

**Figure 3:**
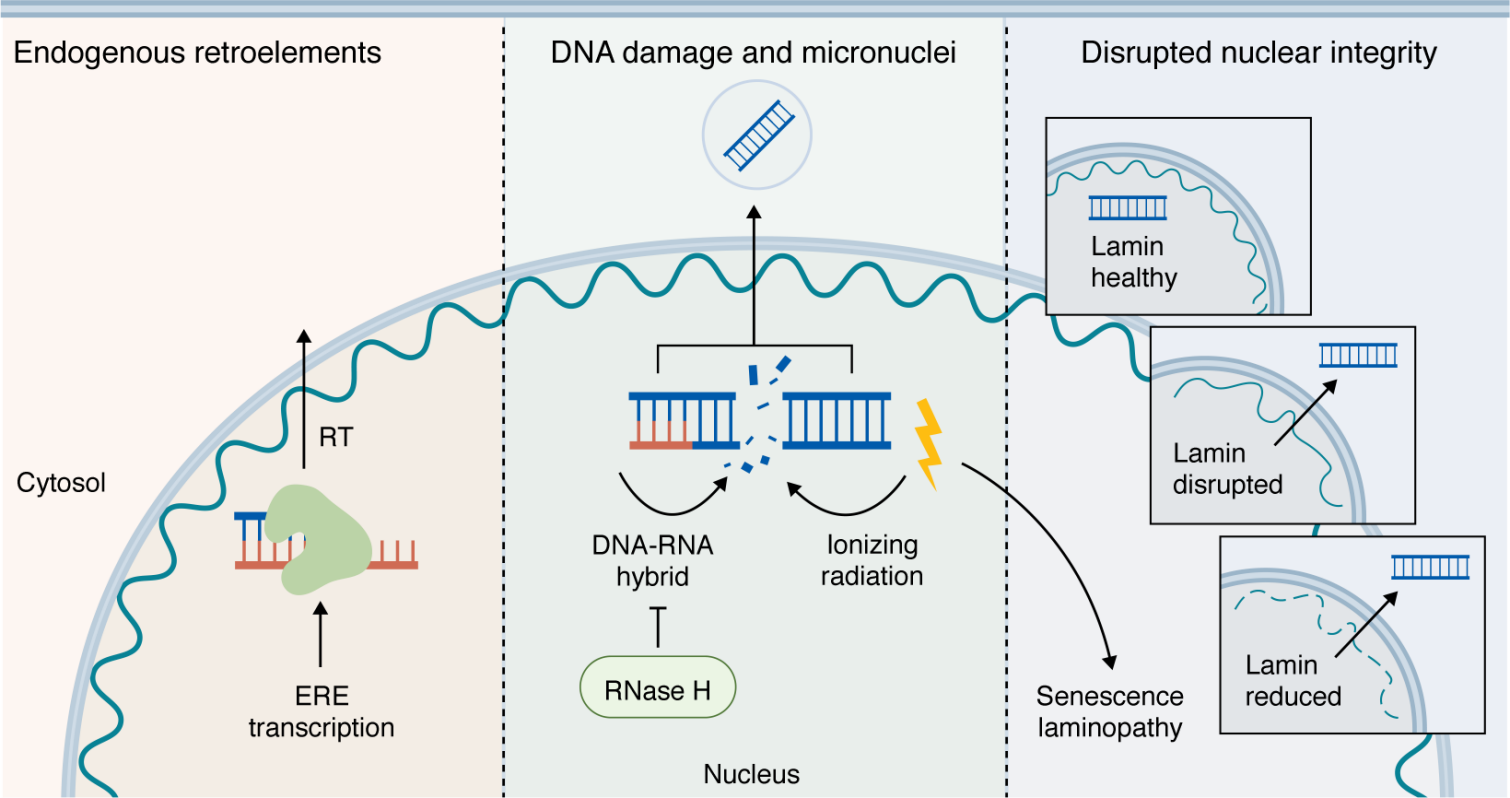
Mechanisms of nuclear DNA release into the cytosol. When TREX1 is absent, self-DNA accumulates in the cytosol, where it is detected by cGAS. Genomic DNA can reach the cytosol through several routes. Cellular stress, DNA damage, or cell death can drive direct release. EREs, which can be activated basally or in response to cytokines, generate RNA templates that undergo reverse transcription (RT), leading to accumulation of ERE-derived DNA copies in the cytosol. DNA damage caused by ionizing radiation or replicative stress can also produce DNA–RNA hybrids when RNase H activity is impaired, promoting the formation of micronuclei (MN). Rupture of MN, which often requires passage through mitosis, releases DNA into the cytosol. Finally, persistent DNA damage can trigger cellular senescence. This occurs in patients with lamin defects, which underlie Hutchinson-Gilford progeria syndrome (HGPS).

There are multiple paths for release of DNA into the cytosol, including breakdown of the nuclear envelope, genomic DNA damage, endogenous retroelements (EREs), and mitochondrial membrane permeabilization (89–94). Dysregulation of these mechanisms can result in an array of human disease, ranging from autoimmunity and autoinflammation to neurodegenerative diseases, all of which intersect with the cGAS-STING pathway (Figure 3).

### Role of the nuclear envelope during cGAS-STING activation.

The nuclear envelope acts as an essential barrier to prevent excessive activation of cGAS and STING. A meshwork of intermediate filaments, called lamins, provide nuclear stiffness and genome organization (95–97). Studies of laminopathies, or diseases of mutated lamins, have also implicated activation of cGAS and STING (98, 99). One nuclear lamin, lamin A (LMNA), is dynamically regulated and stabilized in response to stiff extracellular matrices, protecting against rupture (100, 101). Indeed, lamin levels are reduced by specific cellular stressors (102), and the resulting compromise in nuclear envelope integrity enables escape of chromatin fragments to the cytosol, activating cGAS-STING and promoting the senescence-associated secretory phenotype (SASP) (103, 104). Notably, C-terminal deletions of LMNA cause HGPS, a rare disease marked by premature aging and senescence (105). In the case of HGPS, loss of nuclear integrity is not necessarily attributed to a loss of LMNA, but rather the accumulation of LMNA, which disrupts its nuclear distribution and organization (98). Thus, nuclear envelope integrity functions as a rheostat for cGAS-STING signaling.

### Activation of cGAS and STING in response to DNA damage.

Genomic instability can activate cGAS and STING by producing replication intermediates or chromosomal fragments that leak into the cytosol. For example, in the absence of TREX1, ssDNA intermediates resulting from DNA damage can accumulate in the cytosol to activate cGAS (106, 107). However, cGAS responds less strongly to ssDNA than to dsDNA (108), including in response to dsDNA associated with micronuclei, which are extranuclear structures that encapsulate mis-segregated chromosome fragments (109, 110). When mitosis progresses despite DNA damage, cGAS accumulates at micronuclei, leading to downstream STING signaling (110, 111). During anaphase, the primary substrate for cGAS may be chromatin bridges, potentially explaining the requirement of mitotic progression for cGAS-STING activation (112). However, in some conditions, the presence of TREX1 and nucleosomes can inhibit cGAS activation by micronucleus-derived cytosolic DNA (113–116). In the cytosol, cGAS also acts as a receptor for LC3B, serving to clear micronuclei and to inhibit its own activation in the case of micronuclei rupture (117). Notably, AGS-causing RNase H2 deficiencies also led to the formation of micronuclei, but this phenotype was secondary to DNA damage, likely as a result of impaired ribonucleotide excision repair (118).

Despite its name, TREX1 both promotes and inhibits DNA repair depending on the context (119). In earlier studies, TREX1 was hypothesized to be a proofreading enzyme contributing to base excision repair (BER) in a cell-free system (120), but this role was not detected in *Trex1^–/–^* mice (121). TREX1 was also reported to directly stabilize poly (ADP-ribose) polymerase 1 (PARP1), a ssDNA-binding protein involved in BER (122). TREX1-PARP1 interactions were later verified in HEK293T cells via coimmunoprecipitation–mass spectrometry studies (4). On the other hand, TREX1 may play a role in the SET complex associating with another DNase, NM23-H1, to degrade processed DNA ends required for BER during granzyme A–mediated death (123). Whereas the disease-causing C-terminally truncated form of TREX1 constitutively localizes to the nucleus, WT TREX1 can also localize to the nucleus under certain conditions, such as hydroxyurea treatment, ionizing radiation, and granzyme A–mediated cell death (106, 123). Both WT and RVCL-causing TREX1 mutants have also been reported to inhibit homology-directed repair by degrading 3′ overhangs, further supporting the role of DNA damage in multi-organ degenerative diseases associated with autoimmune features (4).

Originally noted for its capacity to detect cytosolic DNA, more recent evidence indicates that cGAS is a nuclear-cytoplasmic shuttling protein. Removal of the nuclear export signal results in nuclear retention of cGAS and a loss of a type I IFN response upon treatment with immunostimulatory DNA (124). In response to genomic stress, increased nuclear localization of cGAS has been observed (125), reflecting its potential role in the DNA damage response. However, when cGAS interacts with nucleosomes and chromatin, cGAS activation has diminished capacity to signal and induce type I IFN, in part due to steric inhibition of DNA substrates by nucleosomes (114, 116, 126–129).

### Role of EREs in STING-mediated autoinflammation.

ERE cDNA has broadly been considered a driver of AGS pathogenesis, based on work demonstrating accumulation of EREs in TREX1-deficient mice (17). In one early study, reverse transcriptase inhibitor (RTI) treatment of patients with AGS was associated with reduced IFN-γ levels in both blood and cerebrospinal fluid (130). However, RTI therapy is currently not an FDA-approved or accepted part of the clinical care for AGS. Furthermore, *Trex1*^–/–^ mice do not exhibit increased retrotransposition activity, nor do RTIs ameliorate inflammation in *Trex1*^–/–^ animals (131).

LINE1 transposable elements are upregulated in senescent cells, promoting the SASP through cGAS-STING activation (132–134), and cGAS itself may repress LINE1 retrotransposition through TRIM41-mediated degradation of ORF2p (135). This mechanism may be particularly relevant in AGS, where progressive neurological deterioration continues even in patients with controlled IFN signatures. Inflammatory cytokines can reinforce a positive feedback loop via cGAS and STING by further amplifying ERE expression, as observed in the context of human endogenous retrovirus group K induction in M1-polarized macrophages (IFN-γ), as well as astrocytes and neurons (TNF-α) (136, 137). Consistent with this, RTI treatment of senescent cells and aged mice reverses type I IFN activation, further suggesting that the inflammatory cytokine milieu associated with senescence can promote expression of EREs, fortifying a positive feedback loop via cGAS and STING (132).

### Role of mtDNA in cGAS-STING activation.

During apoptosis, mitochondrial outer membrane permeabilization (MOMP) leads to release of mtDNA into the cytosol, resulting in activation of cGAS and STING. However, cytosolic DNA responses are also frequently silenced during apoptosis. For example, type I IFN induction is dampened by caspases, including by caspase-dependent cleavage of cGAS (138–140). Although BAK/BAX-induced MOMP is traditionally known to free cytochrome *c* to the cytosol for apoptosome formation, mtDNA can also be extruded from herniated inner pores (141, 142). In some contexts, mtDNA leakage may be a simple by-product of cytochrome *c* release, requiring cGAS inactivation to prevent excessive inflammation. However, a sublethal MOMP event insufficient to execute cell death has been described to activate cGAS-STING in senescent cells via BAX and BAK macropores (143, 144). In nonapoptotic scenarios, the release of mtDNA might have evolved as a programmed mechanism to trigger activation of cGAS and downstream inflammatory responses (145). Specifically, during pyroptosis, gasdermin D binds cardiolipin and assembles pores in both the outer and inner mitochondrial membranes, facilitating rapid release of mtDNA (146).

BAX/BAK-independent release of mtDNA into the cytosol can occur during mitochondrial inner membrane permeabilization, especially in the context of oxidative stress or NLRP3 inflammasome activation (147, 148). Short mtDNA fragments can escape via two sequential pores: in calcium-triggered mitochondrial permeability transition pores (mPTPs) in the inner membrane, followed by pores formed by VDAC1 (voltage-dependent anion channel 1) oligomerization in the outer membrane (147, 148). For example, VDAC1 inhibition in the MRL/MpJ-Faslpr mouse model ameliorates lupus-like disease and suppresses ISG expression (147). In SLE, mtDNA release may result in downstream inflammation, unshielded by apoptotic caspases (147). Beyond autoimmunity, activation of cGAS and STING by mtDNA can also occur in neurodegenerative diseases. For example, TDP-43 accumulation, a hallmark of amyotrophic lateral sclerosis, drives mPTP-dependent cGAS-STING activation, thereby promoting neurodegeneration (149). Further, packaging of mtDNA is also essential to prevent cGAS-STING activation. Haploinsufficiency of the mtDNA packaging protein transcription factor (transcription factor A, mitochondrial; TFAM) leads to accumulation of cytosolic mtDNA and subsequent cGAS-STING activation (150). The role of TFAM in protecting mtDNA from cytosolic sensing is particularly important in neutrophils, since neutrophils have defective mitophagy, which limits cGAS activation (151, 152). In addition to detecting cytosolic DNA from the mitochondria or the nucleus, extracellular DNA can activate cGAS under conditions where neutrophil extracellular traps are engulfed by phagocytosis (153).

## A DNA damage theory of autoimmunity and autoinflammation

Syndromes linked to DNA damage often exhibit clinical disease phenotypes that are also associated with autoimmunity. The bidirectional crosstalk between DNA damage and nucleic acid–sensing pathways makes it particularly challenging to disentangle mechanisms and pinpoint the initial drivers of disease. This problem is relevant in both common and rare autoimmune diseases, as well as in DNA damage syndromes.

As noted previously, RVCL is one of the most instructive examples of a DNA damage syndrome with features of autoimmunity. This monogenic disease can present with Raynaud’s, autoantibodies, and multi-organ injury, often resulting in the diagnosis being confused with multiple sclerosis or SLE (4, 15, 41, 45, 154). However, patients with RVCL have healthy levels of circulating cytokines, distinguishing RVCL from these classical autoimmune diseases (4, 42). The disconnect between tissue damage and systemic inflammation in RVCL suggests alternative drivers of autoimmune-like organ injury. Given the role of mislocalized TREX1 in causing DNA damage, the combined molecular and clinical features lead us to propose a broader principle: some irreversible clinical manifestations of autoimmunity, including organ damage, may reflect direct injury to the genome, mediated by cytokines in traditional autoimmune diseases and by genotoxic proteins in DNA damage syndromes that have features of autoimmunity. This clinical overlap is also observed in other DNA damage syndromes, such as Fanconi anemia neuroinflammatory syndrome, a close mimic of RVCL (155).

Ataxia-telangiectasia (AT) is another example of a primary DNA damage syndrome with autoimmune features that likely occur through both genomic and metabolic mechanisms. AT is caused by deficiency of the master DNA damage response kinase AT mutated (ATM), yet its pathology extends well beyond impaired double-strand break repair (156–158). ATM also functions in the cytosol as an ROS sensor, activating the pentose phosphate pathway through glucose-6-phosphate dehydrogenase induction, thereby generating the NADPH required for antioxidant defense (159–161). Loss of this function results in persistent oxidative stress that compounds genomic instability. Furthermore, mitochondrial ROS can trigger mitophagy in an ATM-checkpoint kinase 2–dependent manner. In the absence of ATM, this pathway is impaired, promoting mtDNA accumulation and the potential for cGAS-STING activation (151, 162). Indeed, the resulting clinical consequences, such as pulmonary inflammation and neuroinflammation (163, 164), are consistent with the proposition that unresolved DNA damage can contribute to the generation of tissue-specific autoimmune pathology. Thus, AT further underscores the link between DNA damage and inflammation.

In some examples of common autoimmune diseases, oxidative stress and metabolic dysfunction may similarly promote type I IFN signaling. Indeed, oxidative stress and ATP depletion are well-established features of SLE (165, 166), and emerging evidence suggests that mitochondrial dysfunction can drive type I IFN signaling (167, 168). One compelling genetic example involves individuals with loss-of-function mutations in the neutrophil cytosolic factor 1/2, leading to a reduction in NADPH oxidase complex 2 (NOX2) activity in antigen-presenting cells and thereby impairing clearance of oxidized apoptotic debris (167, 168). The resulting accumulation of oxidized cellular material, including mtDNA, leads to activation of cGAS, thus linking defective redox homeostasis directly to the innate immune activation that characterizes SLE (153). Together with the AT data above, this illustrates a recurring theme: when the machinery for managing oxidized or damaged nucleic acids is compromised — whether through loss of TREX1, ATM, or NOX2 — the immunological consequence is autoimmunity.

Cytokines can perpetuate DNA damage through multiple reinforcing pathways, both STING dependent and independent, leading to irreversible organ injury. Transcriptional upregulation of STING increases sensitivity of the pathway (169), and further cGAS and STING activation can amplify nuclear and mitochondrial DNA damage that induces cellular senescence and SASP (54, 170). Downstream of STING, IFN-β treatment was shown to potently induce micronuclei and senescence (171), and ISG15 elevation may contribute through replication fork acceleration and resulting DNA damage (172). Thus, inflammatory mediators not only reflect the response to cellular damage but also actively perpetuate damage via both STING-dependent and -independent pathways. Homeostatic regulators such as TREX1 and cellular antioxidant systems are therefore essential to prevent DNA damage and amplification loops, which likely contribute to irreversible genome damage and associated cellular and organ injury.

## Conclusion

There are roughly 300 million people who develop rare genetic diseases (173). Many of these diseases are disabling and untreatable, and all of these patients deserve the chance to live longer and healthier lives. Their participation in clinical research has led to the discovery of many fundamental biological principles, including the recognition that DNA damage and innate immune activation potentially converge on shared endpoints of organ injury — a theme that may connect RVCL, AGS, SAVI, and COPA syndrome despite their distinct etiologies. Many of these diseases were discovered years ago (SAVI, COPA syndrome), and in some cases even decades ago (RVCL, AGS). For the families with autosomal-dominant syndromes, such as RVCL, the disease has been passed on for generations, and these families are still holding on to hope that the science will lead to real therapies that stop their suffering and save their lives. Thanks to the patients, families, and researchers engaged in this effort, the biomedical research community is on the cusp of first-in-class small molecule therapies that target cGAS (174), STING (175), and TREX1 (176), providing new hope for patients with common and rare diseases alike.

In patients with more common forms of autoimmunity, irreversible organ injury remains a very difficult problem. The current therapeutic focus remains on prevention of damage rather than on restoration of organ function. These same challenges are also shared with many monogenic diseases, including DNA damage syndromes where genomic injury is likely the primary driver of tissue destruction. Matching therapies to specific targets and nodes of dysfunction, whether accumulation of cytosolic DNA or direct genotoxicity, will be essential for improving outcomes in these patient populations. However, looking forward, further elucidation of fundamental biological principles also holds potential to open new frontiers in medicine.

## Author contributions

Writing of the original draft and reviewing and editing were done by DJP, KMJ, JBA, AVF, and JJM.

## Conflict of interest

AVF and JJM are listed as coinventors on separate patents, related to CAR T cells (Boosting Chimeric Antigen Receptor Cells in the Blood; AVF) and small molecule (Small Molecule Inhibitors of Trex1 and Uses Thereof; JJM) and gene therapies (Compositions and Methods for Correction of Trex1 Mutations; JJM).

## Funding support

This work is the result of NIH funding, in whole or in part, and is subject to the NIH Public Access Policy. Through acceptance of this federal funding, the NIH has been given a right to make the work publicly available in PubMed Central.

- Miner laboratory by grants from the NIH (R01AI143982 and R01NS131480).
- Miner laboratory by the Penn Colton Center for Autoimmunity.
- Miner laboratory by charitable gifts from the Clayco Foundation and the Penn RVCL Sisters Fund.

## Figures and Tables

**Table 1:**
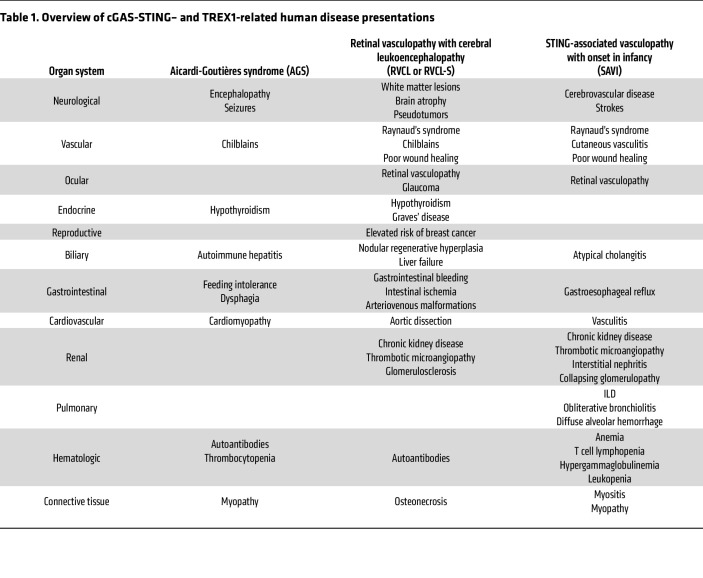
Overview of cGAS-STING– and TREX1-related human disease presentations

## Version 1 06/01/2026

Electronic publication
